# *Rickettsia africae* an Agent of African Tick Bite Fever in Ticks Collected from Domestic Animals in Eastern Cape, South Africa

**DOI:** 10.3390/pathogens9080631

**Published:** 2020-08-02

**Authors:** Benson Chuks Iweriebor, Ayabulela Nqoro, Chikwelu Larry Obi

**Affiliations:** 1 School of Science and Technology, Sefako Makgatho Health Sciences University, Ga-Rankuwa, Pretoria 0208, South Africa; lawrence.obi@smu.ac.za; 2 SAMRC Microbial Water Quality Monitoring Centre, Department of Biochemistry and Microbiology, University of Fort Hare, Alice 5700, South Africa; 201407896@ufh.ac.za; 3 Applied and Environmental Microbiology Research Group (AEMREG), Department of Biochemistry and Microbiology, University of Fort Hare, Alice 5700, South Africa

**Keywords:** ticks, South Africa, spotted fever group rickettsiae, *R. africae*, African tick bite fever

## Abstract

**Background:** Ticks transmit a plethora of pathogens of zoonotic implications. Their distribution, diversity and the pathogens they transmit differ from one ecological location to another. *Rickettsia africae* is the agent of African tick bite fever found in South Africa, a zoonotic infection that is frequently reported among travelers who have visited many sub-Saharan African countries where the pathogen is prevalent. **Methods:** Ticks were collected from domestic animals in Raymond Nkandla Municipality, Eastern Cape, South Africa. The ticks were identified morphologically prior to DNA extraction followed by molecular identification of randomly selected ticks from the morphologically delineated groups. To assess for the presence of tick-borne pathogens belonging to *Rickettsia* spp. by PCR (polymerase chain reaction), we used specific primer pairs targeting the *glt*A, *omp*A and *omp*B genes. The selected amplified ticks, all positive *omp*B and forty three *omp*A amplicons were sequenced in a commercial sequencing facility. The obtained nucleotide sequences were edited and subjected to BLASTn for homology search and phylogenetic analyses were performed with MEGA 7 Version for genetic relationships with curated reference sequences in GenBank. **Results:** A total of 953 ticks collected in the study were delineated into three genera consisting of *Amblyomma*, *Rhipicephalus* and *Hyalomma* in decreasing order of abundance. The presence of rickettsial DNA was detected in 60/953 (6.3%) from the three genera of ticks screened. Genetic analyses of the DNA sequences obtained showed that they have phylogenetic relationship to members of the spotted fever group rickettsiae with *R. africae*, being the predominant SFGR (spotted fever group rickettsiae) detected in the screened ticks. **Conclusion:** This report shows that *R. africae* is the predominant spotted fever group rickettsiae in ticks collected from domestic animals in the study area and the human health impacts are not known.

## 1. Introduction

Tick-borne pathogens have been identified as the etiologic agents of emerging important human diseases especially in many tropical countries in Africa, Asia and South America [1]. In many sub-Saharan Africa countries, these diseases are rampant in rural communities where there are frequent contacts between humans and domestic animals that are hosts to these ticks [1,2]. Some rickettsioses are zoonotic diseases that are caused by some pathogenic *Rickettsia* spp. They are one of the oldest known vector-borne zoonotic diseases whose severity varies from one etiologic agent to another. There are many recognized species of *Rickettsia* that are delineated into four major groups namely; the typhus group consisting of two species which are *R. typhi* and *R. prowazekii*, the spotted fever group which contains many species that are exclusively transmitted to humans through ticks bites, the transitional group comprising of *R. australis*, *R. akari* and *R. felis* that are associated with ticks, mites and fleas and the ancestral group made up of *R. bellii* and R. *canadensis*. Beside these groups, there are also many other *Rickettsia* species that do exist but have not been fully characterized [2,3,4]. The current guidelines for the classification, delineation and description of novel rickettsial isolates are based on the 16S rRNA gene, the differences in nucleotide sequence of *glt*A, *omp*A, *omp*B, and the D genes that encodes for essential proteins in the organisms [5]. The global distribution of tick-borne rickettsioses varies from one region to another as their geographical spread are determined by their tick vectors whose distributions are generally governed by suitable environmental conditions like relative temperatures, humidity and biotopes which varies from one region to the other [6,7]. 

Members of the genera *Amblyomma*, *Hyalomma*, *Rhipicephalus*, *Ixodes*, *Dermacentor* and *Haemaphysalis* are the species of ticks that are generally involved in the transmission of tick-borne rickettsioses in the tropical regions of the world [8,9]. Transmission of tick-borne rickettsioses could be either transstadial or transovarial thus making some tick species such as *Amblyomma* to be a known reservoir of *R. africae* [7]. In the Eastern Cape of South Africa, most common species of ticks are members of the genera; *Amblyomma*, *Rhipicephalus*, *Haemaphysalis*, and *Hyalomma* [8,9] which are well-known vectors of several zoonotic pathogens. While most tick-borne pathogens are linked to known tick vectors, it is possible in some cases for an etiologic agent of a particular disease to be indeterminate. 

Prior to the development of molecular approaches which are highly selective and sensitive in disease diagnoses, several *Rickettsia* spp. had been detected in ticks but their roles in the etiology of diseases in humans were unrecognized. However, in recent times such species that were previously thought innocuous have now been directly linked to the etiology of human diseases [3,10]. *R. africa* is reportedly the most common cause of fever in travelers to Africa, after malaria. The incubation period ranges from 5–10 days and clinical manifestations include the main spot of inoculation, eschar, fever, myalgia, headache, rash, lymphadenitis and in some cases, reactive arthritis [11].

The Eastern Cape of South Africa is predominantly rural with intensive animal husbandry where the animals are kept in close proximity to homes. Couple with this is that there are many game reserves where these animals are in close contact with those in the wild. There exist great possibilities of these domesticated animals being infested with ticks from those originating from the wild thus making the spread of zoonotic pathogens possible in these localities. In addition, with the very high prevalence of HIV/AIDS in these rural communities, the chances of these immunocompromised patients coming down with zoonotic infections are equally very probable as they lack the competent immune system that ordinarily fights off these infections in healthy folks. Besides, most of the spotted fever group rickettsiae (SFGR) infections present with symptoms that are very much similar to flu and could thus be misdiagnosed by clinicians who might not consider them as top priority in diagnoses and treatments due to lack of laboratory diagnosis prior to commencement of treatments. This study therefore, was aimed at epidemiological surveillance for tick-borne *Rickettsia* pathogens in ticks collected from domestic animals in communities which are close to natural game reserves and human habitations in order to assess the likelihood of zoonotic diseases in humans who might be infested with ticks.

## 2. Materials and Methods

### 2.1. Ethical Clearance

The University of Fort Hare Ethics Committee granted the ethical clearance (REC-270710-02-RA) before the commencement of the study. Permission was granted by the farmers before any ticks collection could commence from their animals. Ticks collection was done with help of veterinary personnel and animal health technicians responsible for handling and treating the animals. Ethical clearance certificate (cert number: OBI013) was obtained from University of Fort Hare research and ethics committee (UREC).

### 2.2. Study Area, Tick Collection and Identifications.

The study was conducted from March 2017 to September 2018 in Raymond Mhlaba local Municipality in the Eastern Cape Province. Ticks collection sites and their geographical coordinates are as follows; Debe, with coordinates: 32.836° S, 27.154° E, and Fort Beaufort with coordinates: 32°47’ 0” S, 26°38’0” E. Debe Location shares boundary with Great Fish Natural Reserve which boasts of some wild animals like antelope, boars, giraffe, deer, and others (study site map can be accessed through: https://tse2.mm.bing.net/th?id=OIP.GvQ4IE1x4Slo9oTuUb9EBgHaEk&pid=Api&P=0&w=284&h=176).

### 2.3. Ticks Collection and Studied Animals from Where Ticks Have Been Collected

Ticks were collected manually from 350 domestic animals (goats, sheep, cattle and horses) by using forceps into 50 mL Nalgene tubes that contained 70% ethanol taking precautionary measures to avoid mix-up of samples on the basis of animals and collection sites as tubes were properly labelled and were stored at 4 °C until further processing [8]. All tick species collected were identified using morphological criteria and appropriate taxonomic keys [12]. Ticks genders and feeding states were not taken into consideration as they do not affect their ability to transmit pathogens. 

### 2.4. Total Genomic DNA Extraction from Ticks

Prior to DNA extraction, the preserved specimens were washed with sterile distilled water and left to dry in a sterile Petri dish. This was done in order to remove all the ethanol residues from the tick samples which might negatively affect the downstream PCR reactions. The ticks were singly crushed with sterile glass rod in Petri dishes based on their morphological delineations and processed separately for DNA extraction using ReliaPrep DNA Tissue miniprep system ZYMORESEARCH (Zymo Research Corporation, Irvine, California, CA, USA) Quick DNA Universal Kit according to the manufacturer’s instruction. The adult ticks were processed separately while the nymphs of the same species collected from the same animal were pooled together and processed for DNA extraction. All ticks processing and PCR reactions were performed in biosafety cabinet which were always left sterile overnight using UV light. Absolute precautionary measures were adopted to avoid cross contaminations of reagents, blades and instruments used throughout the process. Commercially certified DNA/RNase free filter barrier tips were used to prevent aerosol contamination while all PCR setup was performed in a hood far from DNA isolation area.

### 2.5. Molecular Identification of Tick and Detection of Bacteria in Ticks

The morphologically identified ticks were confirmed molecularly and profiled for genetic evidence of rickettsial DNAs using the primer pairs in Table 1. These primer pairs have been used in the species identification of *Rickettsia* pathogens as previously reported by Regnery et al. [13], Eremeeva and Raoult [14], and Kollars and Kengluecha [15] as adapted by Williamson et al. [16]. PCR mixtures and cycling profiles were as previously described by the authors.

### 2.6. DNA Sequencing, Sequence Editing and BLASTn Search

The amplified PCR products were sequenced in a commercial sequencing facility using the dideoxynucleotide chain termination approach on an ABI PRISM Genetic Analyzer (ABI Prism310, Applied Biosystems, Foster City, CA, USA). Alignment and sequence editing was achieved using Geneious R10 version. Generated nucleotide sequences were compared with those representative of the rickettsial strains and available in the GenBank database. Rickettsial sequences that had above 97% homology with test samples were used as reference strains for phylogenetic analyses along with other curated representatives of SFGR sequences in GenBank database.

### 2.7. Phylogenetic Analysis

Edited sequences were used to construct a maximum possible phylogenetic tree using Mega 7 version software (PENN, State College, Pennsylvania, PA, USA) with 1000 bootstrap replicate. Phylogenetic trees were constructed using BioEdit Tree Builder. Sequence data sets of positive samples were submitted to NCBI GenBank for accession numbers.

## 3. Results

### 3.1. Tick Prevalence within the Two Study Sites

Out of 953 ticks, 60 (6.3%) resulted positive by targeting the *omp*A and *omp*B genes. Only 43 samples out of the 60 positive *omp*A amplicons were sequenced due to some technical constraints. All the positive *omp*B amplicons were sequenced as well as representatives of tick’s 12S rDNA amplicons while none was sequenced for the *glt*A positive amplicons. The inability to sequence the *glt*A amplicons was due to financial constraints coupled with the fact that it has lower discriminatory power to delineate *Rickettsia* spp. 

Identification methods used to delineate the collected ticks showed that they belonged to three genera, which were *Rhipicephalus*, *Amblyomma* and *Hyalomma* with six different species. Amongst them were *A. hebraeum* 39.14% (n = 373 adult), *R. appendiculatus* 16.4% (n = 156(34) adult), *R. microplus* 13.96% (n = 133 adult), *R. simus.*11.75% (n = 112 adult), *H. truncatum* 9.76% (n = 93 adult) and *Rhipicephalus eversti* 9.02% (n = 86 adult) in decreasing order of their prevalence. *A. hebraeum* was the most prevalent species in both study sites with cattle serving as the common host. 

**Figure 1:**
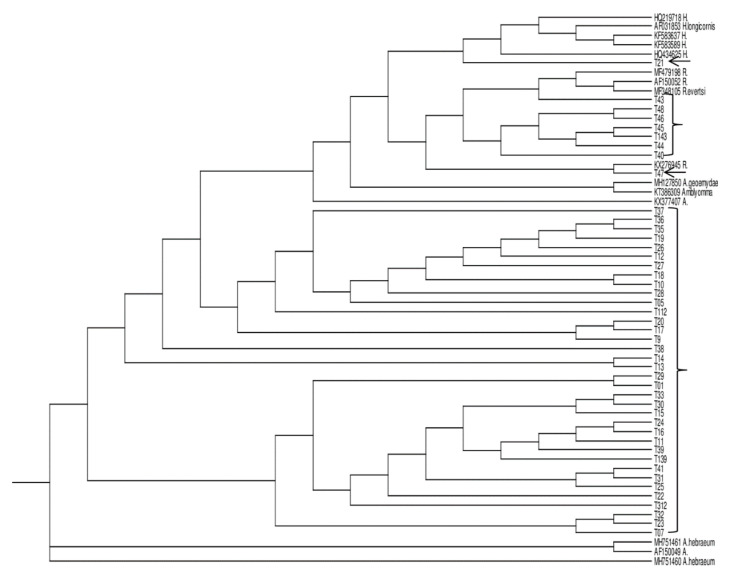
Neighbour-joining rectangular cladogram of 12S mitochondrial rDNA of tick species generated from the study with the reference sequences from GenBank.

Furthermore, BLAST analysis of the edited tick 12S mitochondrial rDNA sequences showed that they all had more than 97% sequence homology with curated ticks’ sequences in GenBank. Molecular confirmation of the representatives of different ticks delineated by morphological methods showed the accuracy of the morphologic classification as shown in Figure 1. The proportions of the different ticks collected in the two study sites are presented in Table 2 while Table 3 is the list of reference sequences obtained from GenBank that were used in the phylogenetic analysis of the ticks’ sequences generated in this study. 

The cladogram was constructed by using the neighbour-joining method in ClustalX 2.1 program (University College Dublin, Dublin, Ireland). Test sequences intermingled with sequences of *Amblyomma*, *Hyalomma*, and *Rhipicephalus* species reference strains obtained from GenBank. All sequenced ticks samples clustered with the three identified genera; *Amblyomma*, *Hyalomma* and *Ripicephalus* reference sequences obtained from GenBank. Test sequences are in bracket and arrowed while the reference sequences are in GenBank accession number.

### 3.2. Prevalence of Rickettsia spp in the Study Sites

Rickettsial DNA fragments were obtained from the three genera of ticks collected in the study sites with more detected in *A. hebreaum.* In addition, a higher proportion of the pathogens (55%) were detected in ticks collected from cattle, 23% prevalence in ticks from goats while that of sheep was 22%. There were no clinical or pathological indicators of disease observed in the animals.

**Figure 2:**
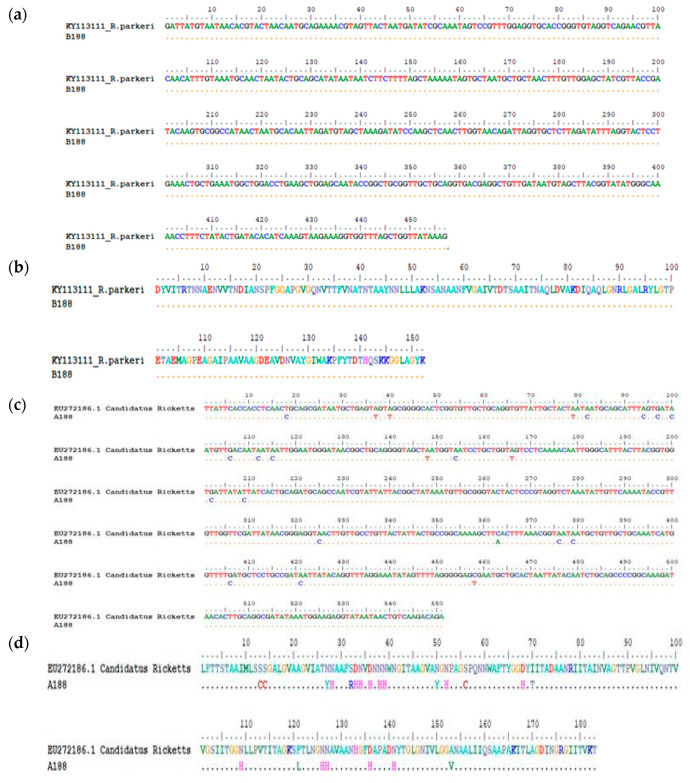
(**a**) Nucleotide sequence alignment of the ompB gene of sample B188 against the homologous reference sequence of *R. parkeri* (KY113111) indicating 100% homology. Reference sequence was obtained based on highest percentage homology that the test sequence has with *R. parkeri* which was obtained through Nucleotide BLAST tool in the GenBank. The dots represent nucleotide similarity of the query sequence with the reference strain. (**b**) Amino acid sequence alignment of the ompB gene of sample B188 against the homologous reference sequence of *R. parkeri* (KY113111) indicating 100% homology. Reference sequence was obtained based on highest percentage homology that the test sequence has with *R. parkeri* which was obtained through Nucleotide BLAST tool in the GenBank. The dots represent amino acid similarity of the query sequence with the reference strain. (**c**) The degree of homology between test sequence A188 and Candidatus Rickettsia (EU272186.1) reference strain: Nucleotide sequence alignment of the ompA gene of sample A188 against the homologous reference sequence of Candidatus Rickettsia sp. (EU272186.1) indicating 95% homology. The dots represent nucleotide similarity of the query sequence with the reference strain. (**d**) Amino acid alignment of the ompA gene of sample A188 against the homologous reference sequence of Candidatus Rickettsia sp. (EU272186.1) indicating 95% homology. The dots represent amino acid similarity of the query sequence with the reference strain.

**Figure 3:**
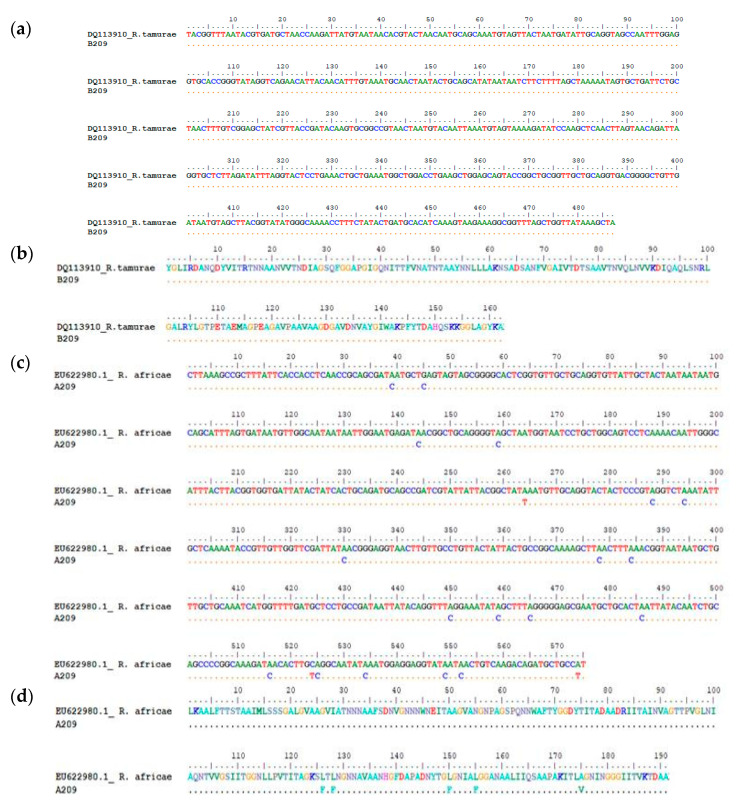
(**a**) Nucleotide sequence alignment of the ompB gene of sample B209 against the homologous reference sequence of *R. tamurae* (DQ113910) indicating 100% homology. Reference sequence was obtained based on highest percentage homology that the test sequence has with *R. tamurae* which was obtained through Nucleotide BLAST tool in the GenBank. The dots represent nucleotide similarity of the query sequence with the reference strain. (**b**) Amino acid sequence alignment of the ompB gene of sample B209 against the homologous reference sequence of *R. tamurae* (DQ113910) indicating 100% homology. Reference sequence was obtained based on highest percentage homology that the test sequence has with *R. tamurae* which was obtained through Nucleotide BLAST tool in the GenBank. The dots represent amino acid similarity of the query sequence with the reference strain. (**c**) The degree of homology between test sequence A209 and *R. africae* (EU622980.1) reference strain: Nucleotide sequence alignment of the *omp*A gene of sample A209 against the homologous reference sequence of *R. africae* (EU622980) indicating high homology with *R. africae* whereas the *omp*B gene of the same sample has 100% identity with *R. tamurae*. The dots represent nucleotide similarity of the query sequence with the reference strain. (**d**) Amino acid sequence alignment of the *omp*A gene of sample A209 against the homologous reference sequence of *R. africae* (EU622980). The dots represent amino acid similarity of the query sequence with the reference strain.

A homology search for the generated *Rickettsia* sequences showed that they had a high sequence similarity of above 97% homology with sequences of *Rickettsia* spp. in GenBank database. Comparison of the 43 *omp*A and 60 *omp*B sequences by BLAST analysis showed that majority of the sequences were ≥98% homologous with *R. africae* while only two samples (B188) and B209 had 99% similarity with *R. parkeri* (KY113111) and 100% homologous with *R. tamurae* (DQ113910), respectively, based on *omp*B BLASTn analysis. However, discordances in homology between *omp*A and *omp*B sequences of the two samples were observed as shown in Figure 2a–d and Figure 3a–d respectively. The *omp*A of sample 188 showed 99% similarity with *Candidatus Rickettsia* (EU272186) while the *omp*B of the same sample was 100% homologous to *R. parkeri* (KY113111) in both amino acid and nucleotide sequences alignments. Similarly, A209 of sample 209 was 100% homologous with *R. africae* (EU622980) while B209 of the same sample was 100% homologous with *R. tamurae* (DQ113910) respectively in both amino acid and nucleotide sequences alignments. 

**Figure 4:**
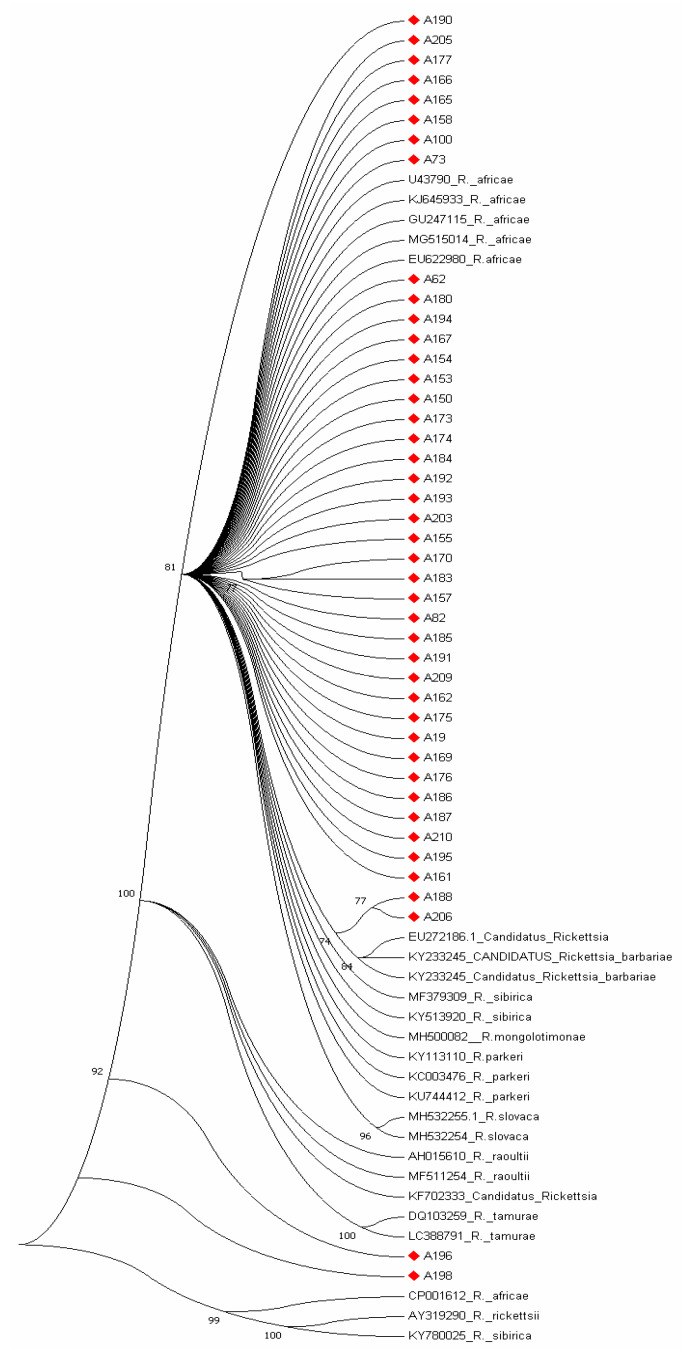
Phylogenetic tree of *omp*A gene sequences in bold red generated from the study with the related reference sequences obtained from NCBI GenBank. The evolutionary history was inferred using the neighbor-joining method [17]. The optimal tree with the sum of branch length = 1.67432413 is shown. The percentage of replicate trees in which the associated taxa clustered together in the bootstrap test (1000 replicates) is shown next to the branches [18]. The evolutionary distances were computed using the p-distance method [19] and are in the units of the number of base differences per site. The analysis involved 67 nucleotide sequences. Codon positions included were 1st + 2nd + 3rd + Noncoding. All positions containing gaps and missing data were eliminated. There were a total of 166 positions in the final dataset. Evolutionary analyses were conducted in MEGA7 [20]. The test sequences denoted diamond red clustered with other *Rickettsia* references.

**Figure 5:**
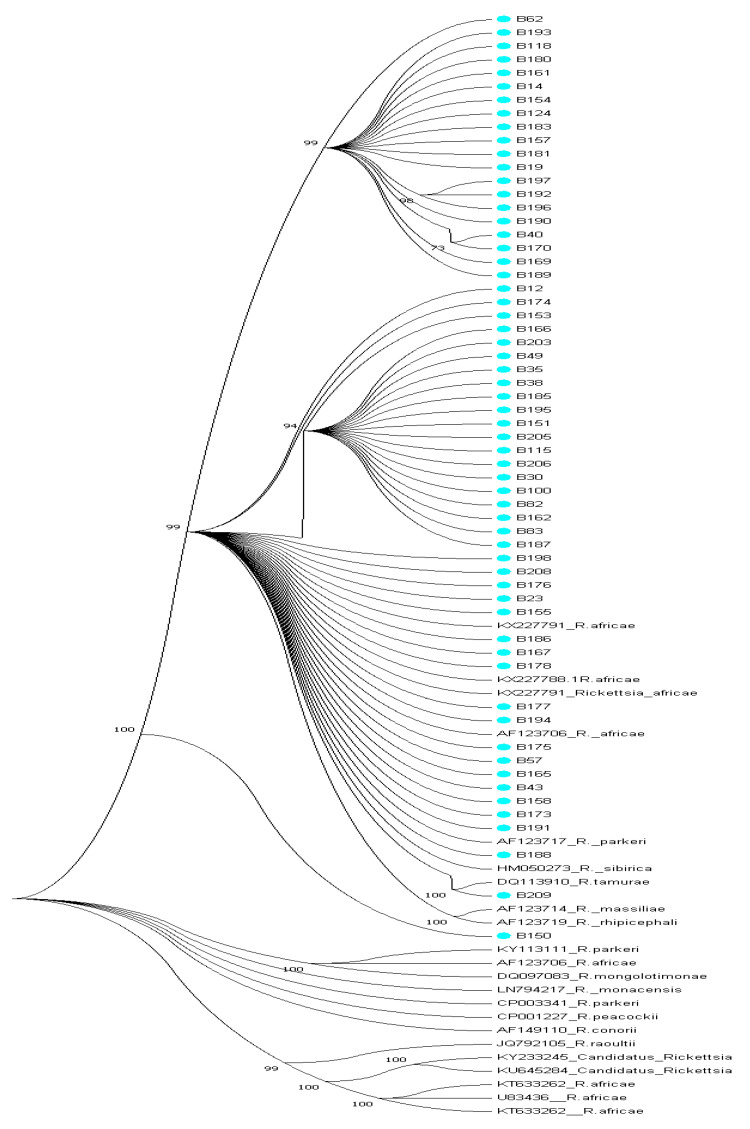
Phylogenetic tree of *omp*B gene sequences in bold generated from the study with the related reference sequences obtained from NCBI GenBank. The evolutionary history was inferred using the neighbor-joining method [17]. The optimal tree with the sum of branch length = 2.89828155 is shown. The percentage of replicate trees in which the associated taxa clustered together in the bootstrap test (1000 replicates) is shown next to the branches [18]. The evolutionary distances were computed using the p-distance method [19] and are in the units of the number of base differences per site. The analysis involved 86 nucleotide sequences. Codon positions included were 1st + 2nd + 3rd +Noncoding. All positions containing gaps and missing data were eliminated. There were a total of 236 positions in the final dataset. Evolutionary analyses were conducted in MEGA7 [20]. All study sequences clustered phylogenetically with *R. africae* sequences from GenBank with the exception of sequences B188 and B209; B188 clustered with *R. parkeri* (AF123717) while sample B209 clustered with *R. tamurae* (DQ113910) with high bootstrap values above 99%. Test sequences are in bold in blue dot.

Reference sequences used for phylogenetic analyses were randomly selected and analyzed with generated study sequences of *omp*A and *omp*B genes. The phylogenetic tree obtained for *ompA* gene showed that test sequences clustered with reference sequences from NCBI GenBank nucleotides database as illustrated in Figure 4. Majority of the *omp*A sequences clustered with *R. africae* (U43790; KJ645933, GU247115, MG515014) with bootstrap values above 81% while sequences A196 and A198 clustered equidistance between *R. tamurae* (LC388791) *R. africae* (CP001612) with 92% bootstrap reliability and A188 and A208 clustered with *Candidatus Rickettsia* EU272186 with 77% bootstrap value as shown in Figure 4 while the phylogenetic tree constructed with *omp*B sequences is shown in Figure 5 with bootstrap values above 70%. Phylogenetic analyses of the *omp*A and *omp*B sequences support the observed discrepancies in homology. Sequence B150 clustered between *R. rhipicephali* (AF123719) and *R. parkeri* (KY113111) phylogenetically but a BLAST search showed it as having close homology with uncultured *Rickettsia* clone and *R. conorii* (FJ015092) by 76% respectively.

### 3.3. GenBank Accession Numbers

Sequences obtained in this study have been deposited in the GenBank database under the following accession numbers: MK347206–MK347212 (tick identification), MK405447–MK405477 (rickettsia *omp*A gene), MK405386–MK405446 (rickettsia *omp*B gene). 

## 4. Discussion

Spotted fever group rickettsiae (SFGR) are emerging infectious diseases with global distribution that are caused by bacteria of the genus *Rickettsia.* These zoonotic diseases are transmitted by ticks’ vectors among wild and domestic animals [21]. Domestic animals and humans are accidental dead end hosts. Currently, the genes encoding two surface proteins, *romp*A and *romp*B are used in the delineation of SFGR members. Fournier et al. [22] proved the usefulness of these genes for taxonomic purposes when they demonstrated the valid description of two new rickettsial species *R. felis* and *R. peacockii.* According to Fournier et al. [22] the presence of an *omp*A gene warrants classification of a rickettsial isolate into the spotted fever group along with *omp*B gene with a homology of 85.8%.

DNAs of *R. africae*, *R. parkeri* and *R. tamurae* belonging to SFGR were detected amongst the ticks genera collected in this study. These three species are closely related and they are the etiologic agents of African and American tick bites fever that are very prevalent in the sub-Saharan African, United States of America and Brazil and rickettsiosis in Japan, respectively [3]. *R. africae,* the etiologic agent of African tick bite fever is generally transmitted by *Amblyomma* ticks to humans and its natural reservoir is wild rodents [3]. 

Even though rickettsial diseases are found globally, there is no one single tick-borne rickettsial diseases that is found all over the world, rather they are restricted to geographical regions, where designated transmitting ticks exist. The majority of the populace living in sub-Saharan Africa might be seropositive for *R. africae*, hence they hardly succumb to African tick bite fever as opposed to travelers to endemic regions of Africa. Sero-prevalence of *R. africae* in Cameroon is between 11.9–51.8% while in Senegal, it ranges between 21.4–51% [23,24,25]. In a group of 940 travelers to South Africa, majority (27%) of them had flu-like symptoms as a result of contracting *R. africae,* the etiologic agent of African tick-bite fever, occasioned by their travels [26] reported a seroprevalence of 51.7% among inpatients identified with febrile fever who were tested for acute SFGR and Typhus Group Rickettsioses TGR in Moshi, Tanzania. 

The majority of data on African tick bite fever ATBF cases, documented to date have been obtained from tourists returning from endemic countries, such as Botswana, South Africa, rural sub-Equatorial Africa, and Zimbabwe [3,26,27,28,29]. Bogovic et al. [3] reported a case of ATBF in a Slovenian traveler returning from Uganda. The 29-year-old man who had no previously known underlying illnesses sought care after returning from a two weeks visit to Uganda for fever, chills, pains and complained of a tick bite a day prior to departing the country. Lorusso et al. [30] were the first people to report about *R. africae* in ticks in Uganda where *R. conorii* had previously been reported by Socolovschi et al. [29] as being prevalent. Similarly, Angerami et al. ([31] reported ATBF in a Brazilian who visited South Africa upon his return to Brazil. He had eschar and symptoms characteristics of ATBF which was confirmed by both immunological and molecular diagnostic methods to be infected by *R. africae.* African tick bite fever have also been reported for the first time by Harrison et al. [32] on an Austrian traveler to East Africa who acquired the disease through tick bite during a visit to Tanzania. ATBF is generally a mild disease and to date, there has not been any reported deaths attributed to infection of *R. africae.* However, just like *R. parkeri* rickettsiosis, the disease caused by *R. africae* is often associated with an inoculation eschar at the spot of attachment of the tick vector. Usually, the symptoms associated with ATBF normally appear many days after the development of the eschar and they are usually that of fever, headache, myalgia, regional lymphadenopathy and generalized rash in about 50% of the cases. 

The *Amblyomma variegatum* tick has been reported to be the vector of *R. africae* with a prevalence of 97.1% in Uganda [33]. However, Waner et al. [34] reported finding the DNA of *R. africae* in *Hyalomma detritum* tick collected from a wild boar in Israel indicating that the spotted fever group rickettsia is not limited in distribution to the African continent nor to a given host tick. Similarly, Yssouf et al. [35] reported the detection of *R. africae* in 90% of *A. variegatum*, 1% of *R. appendiculatus* and 2.7% of *Rhipicephalus (Boophilus) microplus* in study ticks collected from locally domesticated animals in the Union of the Comoros, as well as in 77.14% in *A. variegatum* ticks obtained from cattle imported into the country. In addition, Maina et al. [36] reported the detection of *R. africae*–genotype DNA in 92.6% of adult *A. variegatum* ticks collected from domestic ruminants in Kenya even though they found no evidence of the pathogen in blood specimens in the domestic animals sampled. *R. africae* genetic materials have been detected by PCR from different species of ticks belonging to *Amblyomma, Rhipicephalus, Hyalomma* genera in several African countries such as Mali, Senegal, Guinea, Liberia, Sudan, Democratic Republic of Congo, Cameroon, Nigeria, Niger, Kenya, Cote di’voire and Burundi [1,3,37] and these reports are in consonant with our finding as the DNA of *R. africae* was detected in the different genera of ticks that we assessed.

*R. parkeri*, a member of the spotted fever group rickettsia, is the etiologic agent of American tick bite fever that is prevalent in the South and North American continents and it is transmitted by the Amblyomma species. The spotted fever disease associated with the organism is characterized by eschar related ailments in humans which are similar to symptoms of Rocky Mountain spotted fever. The index rickettsiosis spotted fever case caused by *R. parkeri* was first recognized by Paddock et al. [38] and ever since then; numerous cases have been identified and reported in many southeastern states of the USA [39,40]. Cowdry, [41], was the first to describe the finding of the organism in the tissues and eggs of female *A. maculatum* ticks that were collected in Jackson County, Missouri. However, Parker et al. [42] isolated the organism for the first time from Gulf Coast ticks that were collected in South eastern Texas and ever since then, *R. parkeri* a SFG rickettsiae has been frequently detected in *A. maculatum. R. parkeri* has however been detected in other tick species other than *A. maculatum* as Williamson et al. [16] reported the detection of its DNA in *D. variabilis* in ticks removed from persons in Texas, USA. *R. parkeri* infections in dogs and cows have been described in southeastern United States. Infection of humans by *R. parkeri* in most cases is associated with a necrotic eschar at the point of inoculation after several days of an infected tick bite and it is usually with a low grade to moderate fever that is very similar to Rocky Mountain spotted fever (RMSF)though less in severity. Some of the symptoms associated with *R. parkeri* rickettsioses are fever, inoculation eschar, macules or papules rashes, vesicles or pustules, petechiae on palms or soles, headache, myalgias, sore throat, lymphadenopathy, diarrhea, nausea or vomiting [40]. No case of *R. parkeri* rickettsiosis has however been reported in Central America but *A. maculatum* is widely distributed throughout the region and a mild eschar-related rickettsiosis that is very akin to *R. parkeri* rickettsiosis has been reported in a traveler who returned from Honduras [43]. Since the first human disease case caused by *R. parkeri* was documented by Paddock et al. [38] numerous cases of rickettsioses caused by *R. parkeri* have been reported among persons residing in the ecological range of the vector tick, *A. maculatum*, in the USA. Infections and eschar associated illness with *R. parkeri* have been frequently reported in several Latin American countries such as Argentina, Brazil, and Uruguay, and the organism has been detected in *A. triste* ticks [44,45]. For the first time, here we report the detection of genetic material of *R. parkeri* in the African continent and the epidemiological implications are not well known. However, because it has been documented as a human pathogen, its involvement in human cases in the study sites may not be unlikely as it may probably have gone undetected. We observed discordant phylogenetic assignments of the *omp*A and *omp*B genes of sample 188 as they were found to cluster with *Candidatus_Rickettsia* EU27216.1 and *R. parkeri* KY113110 respectively in Figure 4 and Figure 5 and this was shown to be so with nucleotide and amino acid alignments as shown in Figure 2a–d. 

*Rickettsia* sp. strain Ga-Seema is an incompletely described rickettsial that was detected from three fed adult male *Rhipicephalus simus* ticks collected from two donkeys in 2014 in Hlahlagane, Limpopo Province, South Africa by Halajian et al. [46] which has not been reported previously and its pathogenic potential is currently unknown. 

*R. tamurae* infection according to Imaokaa et al. [47] is associated with symptoms such as mild local inflammatory signs like swellings, erythema, redness heat and pain. Symptoms of *R. tamurae* infection mimics cellulitis with increased serum titers of antibody against the organism [48]. Unlike most SFGR, infections with *R. tamurae* are not associated with high fever, generalized rash, lymphadenopathy as it is often seen in other spotted fever rickettsioses. *R.tamurae* was first isolated from *A. testudinarium* ticks in Japan and has the wild boar and domestic pigs as it primary host although it can also infest deer, cattle, other ungulates and domestic livestock as well as humans [47,48,49]. *R. tamurae* has been isolated from the skin biopsy specimen from wild boars and also in ticks [47]. It was previously thought to be non-pathogenic to humans until it was reported in human cases in Japan as well as in Laos where its involvement in spotted fever case was documented after a patient tested seropositive for the organism [50]. Phylogenetic analyses of the *omp*A and *omp*B sequences of sample 209 assigned them as *R. africae* and *R. tamurae,* respectively, and homology search confirmed that *omp*A sequence is *R. africae* while the *omp*B sequence had 100% similarity with *R. tamurae*. We performed nucleotide and amino acid sequences alignments with the two sequences as shown in Figure 3a–d, the *omp*B showed complete homology with *R. tamurae* indicating that sample B209 is most highly *R. tamurae* in the *omp*B gene region while the *omp*A was closely related with *R. africae*. We are not sure if recombination did occur in the two genes in question. Further study, like full genome sequencing, is needed to elucidate this observation. *R. tamurae* has been associated with different *Amblyomma* spp. as reported by Blanco et al. [51] who detected the pathogen in screened nymphs of *A. ovale* tick collected from small mammals such as wild rodents and marsupials in Brazil while a recent report stated its detection in a *Haemaphysalis megaspinosa* tick [51]. However, this is the first report of *R. tamurae-*like pathogen, the agent of SFG rickettsiosis in Japan and some Far East Asian countries in *A. variegatum* tick collected from cattle in the African continent. 

## 5. Conclusions and Recommendations

We have reported on the detection of tick-borne rickettsia pathogens which are the etiologic agents of spotted fever group rickettsioses. The findings of the DNA of *R. africae, R. parkeri* and *R. tamurae* in the three genera of ticks collected in this study are of clinico-epidemiological significance and merits further investigations. The discordance of the *omp*A and *omp*B gene fragments of the same samples is noteworthy and a full genome sequencing or a complete *omp*A and *omp*B is, therefore, warranted. Usually, the SFGR are not considered in the diagnosis and treatment of patients with signs and symptoms of fever in these rural communities. This data is therefore a wakeup call on health care personnel to consider infections caused by these pathogens as they may probably be responsible for the flulike symptoms presented by their patients. Education and awareness campaigns in rural communities on the implications of tick bites, control strategies for ticks, including seeking medical attention for tick bites, should be mounted. 

## Endnote

GenBank Accession numbers of Rickettsia reference sequences used in phylogenetic analyses of the ompA and ompB genesOMPBDQ113910_R.tamurae, CP003341_R.parkeri, AF123706_R.africae, KX227791_R.africae, AF149110_R.conorii, KX227788_R.africae, KY113111_R.parkeri, LN794217_R. monacensis, CP001227_R.peacockii, JQ792105_R.raoultii, U83436_R.africae, KY233245_Candidatus Rickettsia, AF123714_R. massiliae, AF123719_R. rhipicephali, HM050273_R. sibirica, KU645284_Candidatus Rickettsia, KT633262_R.africae, DQ097083_R. mongolotimonae, KX227791_R. africae, KT633262.1 R.africae, KY113111_R_ parkeri, AF123706_R. africae, AF123717_ R. parkeriOmpACP001612_R. africae, AH015610_R. raoultii, AY319290_R. rickettsia, KF702333_Candidatus Rickettsia, KY780025_R. sibirica, EU272186_ Candidatus Rickettsia, MF511254_R. raoultii, MH500082 _R. mongolotimonae, MH532255_R.slovaca, KY113110_R.parkeri, KY233245_CANDIDATUS Rickettsia barbariae, MF379309_R. sibirica, KY513920_R. sibirica, EU622980_R.africae, MH532254_R.slovaca, MG515014_R. africae GU247115_R. africae, KJ645933_R. africae, U43790_R. africae, DQ103259_R. tamurae, LC388791_R. tamurae, KC003476_R. parkeri, KU744412_R. parkeri

## Figures and Tables

**Table 1:** Primer sequences used in the molecular identification of ticks and *Rickettsia* spp.

	Primer Name	Gene Primer Sequence (5′ to 3′)	Amplicon bp	TM	Ref
Tick DNA	85F	*12S* TTAAGCTTTTCAGAGGAATTTGCTC	110	54.0	[12]
	225R	*12S TTTWWGCTGCACCTTGACTTAA*		52.7	
Rickettsia spp.	Rr.190 70P	*rompA* ATGGCGAATATTTCTCCAAAA	610	52.5	[13]
	Rr.190 602N	*rompA AGTGCAGCATTCGCTCCCCCT*		64.9	
	BG1-21	*rompB GGCAATTAATATCGCTGACGG*	511	55.6	[14]
	BG2-20	*rompB GCATCTGCACTAGCACTTTC*		55.2	
	RrCS372	*gltA TTTGTAGCTCTTCTCATCCTATGGC*	410	59.0	[15]
	RrCS989	*gltA CCAAGTTCCTTTAATACTTCTTTGC*		57.4	

Bp = base pair, spp = species TM = melting temperature.

**Table 2:** Proportion and distribution of collected tick species in Debe Location and Fort Beaufort, geographical coordinates: 32.836° S, 27.154° E, coordinates 32°47′0″ S, 26°38′0″ E.

Animals	Tick Species	Developmental Stage	Number of Ticks	Rickettisa Positive Samples
CATTLE	*R. eversti*	Adult	77	5
	*A. hebraeum*	Adult	170	25
	*R. microplus*	Adult	27	
	*R. appendiculatus*	Adult	94	3
	*R. simus*	Adult	67	
	*H. truncatum*	Adult	55	
GOAT	*R. eversti*	Adult	0	
	*A. hebraeum*	Adult	133	12
	*R. microplus*	Nymph	79	
	*R. simus*	Adult	24	
	*H. truncatum*	Adult	20	
	*R. appendiculatus*	Adult	62	2
SHEEP	*R. eversti*	Adult	9	9
	*A. hebraeum*	Adult	65	4
	*R. microplus*	Adult	27	
	*R. simus*	Adult	21	
	*H. truncatum*	Adult	18	
HORSE	*A. hebraeum*	Adult	25	

**Table 3:** Reference tick strains used in phylogenetic analysis.

Strain Accession Number	Species	Geographical Origin
KU284929	*A. trigrinum*	Brazil
KU284920	*A. triste*	Uruguay
KU284864	*A. parvitarsum*	Argentina
KY676832	*R. annulatus*	Israel
KY676839	*R. australis*	South Africa
MF479198	*R. evertsi*	DRC
AF150043	*Boophilus*	Jordan
EU921766	*R. microplus*	Mozambique
KU568502	*R. geigyi*	Guinea-Bissau
MK332391	*R. microplus*	Uganda
MG076938	*A. maculatum*	Mexico
KX377407	*A. gemma*	Ethiopia
AF150049	*A. hebraeum*	Zimbabwe
AF031865	*R. punctatus*	Australia

## Data Availability

The data that support the findings of this study are openly available in [GenBank] at https://www.ncbi.nlm.nih.gov/nuccore, MK347206–MK347212 (tick identification), MK405447–MK405477 (rickettsia *omp*A gene), and MK405386–MK405446 (rickettsia *omp*B gene).
